# The Effect of Kefir Supplementation on Improving Human Endurance Exercise Performance and Antifatigue

**DOI:** 10.3390/metabo11030136

**Published:** 2021-02-25

**Authors:** Mon-Chien Lee, Wei-Lun Jhang, Chia-Chia Lee, Nai-Wen Kan, Yi-Ju Hsu, Chin-Shan Ho, Chun-Hao Chang, Yi-Chen Cheng, Jin-Seng Lin, Chi-Chang Huang

**Affiliations:** 1 Graduate Institute of Sports Science, National Taiwan Sport University, Taoyuan 333325, Taiwan; 1061304@ntsu.edu.tw (M.-C.L.); 1081303@ntsu.edu.tw (W.-L.J.); ruby780202@ntsu.edu.tw (Y.-J.H.); kilmur23@ntsu.edu.tw (C.-S.H.); 1031301@ntsu.edu.tw (C.-H.C.); 2 Culture Collection & Research Institute, Synbio Tech Incorporation, Kaohsiung 821, Taiwan; cclee@synbiotech.com.tw (C.-C.L.); yccheng@synbiotech.com.tw (Y.-C.C.); 3 Center for General Education, Taipei Medical University, Taipei 11031, Taiwan; kevinkan@tmu.edu.tw

**Keywords:** kefir, exercise performance, fatigue

## Abstract

Kefir is an acidic, carbonated, and fermented dairy product produced by fermenting milk with kefir grains. The *Lactobacillus* species constitutes an important part of kefir grains. In a previous animal study, kefir effectively improved exercise performance and had anti-fatigue effects. The purpose of this research was to explore the benefits of applying kefir to improve exercise performance, reduce fatigue, and improve physiological adaptability in humans. The test used a double-blind crossover design and supplementation for 28 days. Sixteen 20–30 year-old subjects were divided into two groups in a balanced order according to each individual’s initial maximal oxygen uptake and were assigned to receive a placebo (equal flavor, equal calories, 20 g/day) or SYNKEFIR™ (20 g/day) every morning. After the intervention, there were 28 days of wash-out, during which time the subjects did not receive further interventions. After supplementation with SYNKEFIR™, the exercise time to exhaustion was significantly greater than that before ingestion (*p* = 0.0001) and higher than that in the Placebo group by 1.29-fold (*p* = 0.0004). In addition, compared with the Placebo group, the SYNKEFIR™ administration group had significantly lower lactate levels in the exercise and recovery (*p* < 0.05). However, no significant difference was observed in the changes in the gut microbiota. Although no significant changes in body composition were found, SYNKEFIR™ did not cause adverse reactions or harm to the participants’ bodies. In summary, 28 days of supplementation with SYNKEFIR™ significantly improved exercise performance, reduced the production of lactic acid after exercise, and accelerated recovery while also not causing any adverse reactions.

## 1. Introduction

During long-term or high-intensity exercise, considerable energy is consumed, when energy cannot maintain a stable supply, it may cause fatigue and reduce exercise performance [1]. Fatigue can be divided into central fatigue and peripheral fatigue, the latter of which is defined as the inability to maintain power output and strength, thereby impairing body functions [2]. Physical fatigue is a complex physiological process. The physical and psychological effects of exercise depend on the type, intensity, duration, and energy expenditure of the exercise [3]. These factors are used to describe the decline in bodily function and the actual/perceived difficulties associated with tasks or increased exercise [4]. The most well-known fatigue mechanism is related to the availability of metabolic fuel and waste accumulation. For example, the excessive production and accumulation of metabolites such as lactic acid and urea nitrogen can lead to muscle failure [5]. Many studies have shown that after much exercise, the role of inflammation-related metabolites (such as creatine kinase (CK) and ammonia (NH_3_)) in muscle damage is fully realized [1]. In addition, biochemical indicators, including lactic acid, have been used to assess skeletal muscle fatigue, showing that the serum urea nitrogen (BUN) levels increased, and the glucose (GLU) levels decreased [6].

In recent years, the relationship between gut microbes and energy utilization and exercise performance has gradually attracted attention. The link between exercise and the composition of the gut microbiota seems to be two-way [7]. Not only can exercise training cause changes in the flora and increase its diversity and abundance [8], but the proportion and timing of the dietary intake are also important factors affecting the gut microbiota and proportions [9]. The composition and metabolic activity of the gut microbiota promote digestion and food absorption to produce host energy and also have a great impact on athletes’ energy consumption and exercise performance [10]. In the colon, complex carbohydrates are digested and then fermented into short-chain fatty acids (SCFAs), such as acetate, propionate, and n-butyric acid, which are important sources of gut microbes that provide energy for the body and exercise [11]. In addition, the gut microbiota can also promote metabolism and bile acid synthesis, thereby directly changing mitochondrial biogenesis, inflammation and intestinal barrier function, and, combined with SCFA, can improve energy efficiency and fatigue resistance, thereby improving exercise performance [12].

Kefir is an acidic, carbonated, and fermented dairy product produced by fermenting milk with kefir grains, which are composed of various powders and bacteria [13]. Originating in the Balkans, Eastern Europe, and the Caucasus [14], kefir is traditionally made with milk, but it can also be made with milk from other sources, e.g., goat, sheep, buffalo, and soy milk [15]. Kefir is considered to be a probiotic, among them, *Lactobacillus* species is an important part of kefir grains and constitutes the vast majority of relevant bacterial species, such as *L. paracasei*, *L. casei*, *L. kefiranofaciens*, and *Lactococcus lactis* [16]. These bacteria have potential health benefits, including lowering inflammation levels, exerting anti-cancer effects, lowering serum cholesterol levels, improving digestion and intestinal health, reducing high blood pressure, and regulating active oxygen [17]. Previous studies have shown that supplementation with kefir can effectively improve exercise endurance performance and reduce the serum C-reactive protein (CRP) [18]. In addition, animal experiments have shown that kefir can not only improve exercise endurance but can also reduce the biochemical parameters of fatigue and increase glycogen content [19]. However, more research is needed to confirm the substantial benefits of kefir for humans. Therefore, we investigated whether kefir beverage can promote changes in the gut microbiota, improve exercise endurance performance, and show antifatigue function via regulating key indicators (e.g., lactate, NH_3_, CK) during and after exercise

## 2. Results

### 2.1. Subject’s Basic Biochemical Parameters and Hematology

The 16 recruited subjects met the inclusion criteria, and no one withdrew from the trial. Table 1 outlines the serum biochemical data before placebo or SYNKEFIR™ intervention. Only one item of the TC data in the Placebo group was significantly higher than that in the SYNKEFIR™ group (*p* = 0.0443), while the other items were not significantly different.

Hematology tests were performed before the intervention. We also calculated the ratio of the neutrophil count to the lymphocyte count (NLR) and the ratio of the platelet count to the lymphocyte count (PLR) according to the indicated reference indicators. All data were in a normal range, with no significant difference between the Placebo and SYNKEFIR™ groups (Table 2).

### 2.2. Effects of SYNKEFIR™ Supplementation on Endurance Performance

**Figure 1:**
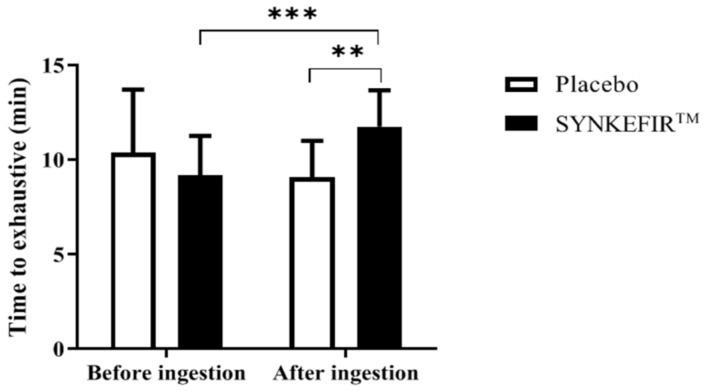
Effects of SYNKEFIR™ supplementation on the time to exhaustion. Data are shown as the mean ± SD, *n* = 16 subjects/group. Intergroup differences were analyzed by two-way repeated measures ANOVA post hoc Bonferroni test. ** *p* < 0.01, *** *p* < 0.001.

As shown in Figure 1, before ingestion, the Placebo group and SYNKEFIR™ group’s exhaustion test times were 10.38 ± 3.34 and 9.19 ± 2.08 min, respectively. There was no significant difference between the two groups. After 4 weeks of ingestion, the Placebo group and SYNKEFIR™ group’s exhaustion test times were 9.08 ± 1.92 and 11.76 ± 1.92 min, respectively. Compared with the Placebo group, the SYNKEFIR™ group’s time was significantly greater by 1.29-fold (*p* = 0.0048). In addition, compared with the period before ingestion, only the SYNKEFIR™ group significantly improved their exhaustion test time after ingestion (*p* = 0.0008).

### 2.3. Effect of SYNKEFIR™ Supplementation on Physiological Adaptation and Biochemical Indices

**Figure 2:**
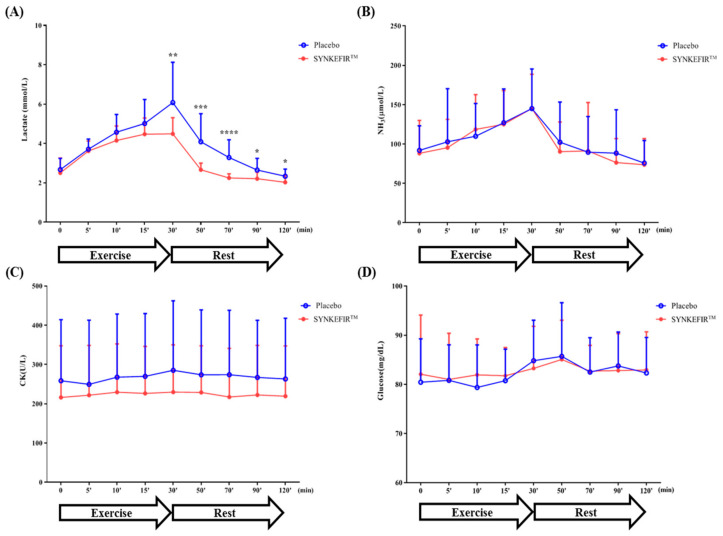
Effects of SYNKEFIR™ supplementation on the (**A**) lactate, (**B**) NH_3_, (**C**) CK, and (**D**) glucose serum levels during and after exercise. Data are shown as the mean ± SD. Statistical differences between groups at same time point were analyzed using Student’s unpaired *t*-test. * *p* < 0.05, ** *p* < 0.01, *** *p* < 0.001, **** *p* < 0.0001.

The serum lactate level increased with the extension of exercise time until reaching 30 min of exercise intervention and gradually decreased to the basic level during the recovery phase. Before administration, the changes in circulating lactic acid during the measurement period were similar in each group. However, compared with the Placebo group, the SYNKEFIR™ administration group had significantly reduced lactate level accumulation in the exercise phase (time point: E30) (*p* = 0.0094) and significantly improved accumulation in the recovery phase [time point: R20 (*p* = 0.0012), R40 (*p* = 0.0003), R60 (*p* = 0.0159), and R90 (*p* = 0.0258)] (Figure 2A).

### 2.4. Effect of SYNKEFIR™ Supplementation on Exercise/Rest Biochemical Parameters and Hematology

Four weeks after the SYNKEFIR™ intervention, a 60% VO_2max_ exercise challenge was performed, and blood was collected for a biochemical numerical analysis before exercise (0′), exercising for 30 min (E30′), and 60 min after exercising for 30 min (R60′). There were no significant differences between the Placebo and SYKKEFIR groups in the liver function indicators, kidney function indicators, and blood lipid metabolism (Table 3).

In addition, in terms of the hematology examination and blood count, there were no significant differences between the Placebo group and the SYNKEFIR™ group at each time point (Table 4).

### 2.5. Effect of SYNKEFIR™ Supplementation on Body Composition

To determine whether supplementation with SYNKEFIR™ had an effect on changes in body composition, we used InBody 570 to measure the body composition before and after intervention. As shown in Table 5, after four weeks of intervention, there was no significant difference on body weight, body mass index (BMI), fat mass, and muscle mass between Placebo and SYNKEFIR™ groups. In addition, the change of body composition between before and after intervention was calculated. The changes of body weight, body mass index (BMI), fat mass, and muscle mass were not significantly different between the Placebo and SYNKEFIR™ groups.

### 2.6. Effect of SYNKEFIR™ Supplementation on the Gut Microbiota

**Figure 3:**
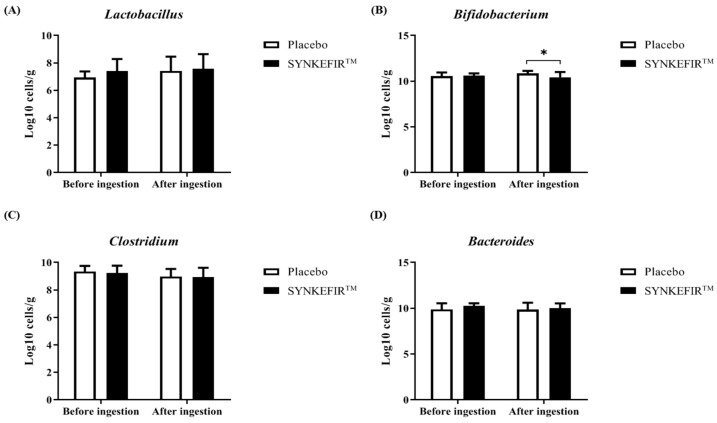
The levels of (**A**) *Lactobacillus* spp., (**B**) *Bifidobacterium* spp., (**C**) *Clostridium* spp., and (**D**) *Bacteroides* spp. were analyzed using qPCR method in fecal samples from subjects with Placebo or SYNKEFIR™ supplementation. Data are shown as the mean ± SD. Intergroup differences were analyzed by two-way repeated measures ANOVA post hoc Bonferroni test. * *p* < 0.05.

As shown in Figure 3, only four genera have been taken into account for the gut microbiota analyses. The qPCR method was used for the identification and quantification of gut microbiota. After four weeks of intervention, bacteria counts of *Lactobacillus* spp., *Clostridium* spp., and *Bacteroides* spp. were not significantly different between Placebo and SYNKEFIR™ groups, but *Bifidobacterium* spp. counts in SYNKEFIR™ groups was significantly decreased compared to Placebo group.

## 3. Discussion

In previous studies, kefir was shown to effectively reduce fatigue after exercise and promote the improvement of exercise performance, which might be affected by changes in the gut microbiota [19]. In the current experiment, we recruited untrained and healthy people to study their sports performance, physiological fitness, inflammation, body composition, and safety to further understand and verify the biological application and health promotion effect of SYNKEFIR™ in sports science.

In recent years, the relationship between gut microbes and exercise performance has gradually attracted attention. Several studies have noted that compared with sedentary people, athletes’ gut microbes are more abundant and diverse [8,20]. In addition, microbes can enhance athletes’ metabolic pathways (such as amino acid and antibiotic biosynthesis and carbohydrate metabolism) and fecal metabolites (such as SCFA produced by microorganisms, such as acetic acid, propionic acid, and butyric acid) to promote health and exercise performance [20]. The metabolism of SCFA seems to be an important factor in increasing the energy required for exercise and improving exercise endurance. Acetate and propionate are used as substrates for energy metabolism and are transported to liver cells for gluconeogenesis [21]. Butyrate is transported to the mitochondria under aerobic conditions and recombined into acetyl-CoA, which enters the Krebs cycle to form NADH and then enters the electron transport chain to produce ATP and CO_2_ [22], which acts in the liver and muscle cells at the same time. SCFA seems to be able to suppress inflammatory cytokines and reduce redox reactions by regulating the function and metastasis of neutrophils, in addition to reducing fatigue by reducing the permeability of the colonic mucosa [14,23]. Therefore, probiotics/prebiotic-related products can increase intestinal SCFA content and are thus being used as a new generation of sports nutrition supplements. Previous studies showed that *B. longum* subsp. *longum* from Olympic weightlifters not only promoted athletic performance and reduced post-exercise fatigue in animal experiments but also improved the exercise endurance performance of professional medium-distance runners [24]. Kefir grains contain lactic acid bacteria, acetic bacteria, and yeasts. Among them, lactic acid bacteria are considered to be very beneficial to the human body and one of the most common probiotic strains. Chen et al. and Huang et al. reported that 6 weeks of supplementation with *L. plantarum* TWK10 can effectively reduce the biochemical value of fatigue after exercise, such as the lactate index. In addition, TWK10 had a significant effect on improving exercise endurance performance [25,26]. Another study focused on female swimmers who consumed yogurt containing *L. acidophilus*, *L. delbrueckii*, *B. bifidum*, *and Streptococcus thermophilus* for more than 2 months. The results showed that compared with the Placebo group, the yogurt-drink-supplemented group significantly increased their maximum oxygen uptake, but had no impact on the 400-m swimming time [27]. However, in current study was shown that after untrained healthy subjects supplemented with a kefir drink for 28 days, the endurance time was significantly improved compared to that before supplementation and was also significantly greater than that in the Placebo group (Figure 1).

In addition to improved exercise endurance, kefir seems to play an important role in reducing the generation of fatigue and accelerating the recovery of fatigue after exercise. Although it had no significant effect on post-exercise fatigue biochemical indicators such as NH_3_, CK, and glucose, supplementation with kefir can effectively reduce blood lactic acid after exercise and rest (Figure 2). The production of lactic acid during muscle-based exercise can cause major changes in the homeostasis of muscle cells and the entire body [28]. Hydrogen ions are produced together with lactic acid, which might be due to the dissociation of lactic acid [29]. The decrease of the pH in blood and muscle tissue can inhibit muscular contractions and glycolysis, which affects not only the metabolic process but also the contraction process of muscle cells, as well as causing various harmful biochemical, metabolic, and physiological side effects [30]. In addition, lactic acid is also considered to be an aerobic metabolite. When oxygen is sufficient, it can be used by the skeletal muscle and the heart and may contribute to the formation of acetyl-CoA [31]. Most of the lactic acid derived from the muscle is transported to the liver, where glucose is synthesized through gluconeogenesis. Glucose passes through the bloodstream and reaches the muscles, where it is used as a metabolite for glycolysis (Cory Cycle) [5]. Supplementation with probiotics can accelerate the conversion of lactic acid produced during exercise into butyrate and then into acetyl-CoA, which is used to generate ATP in the Krebs cycle [32]. Past studies have confirmed that supplementation with SYNKEFIR™ can help subjects use lactic acid to produce SCFA, thereby increasing the utilization of nutrients, improving exercise performance, and reducing fatigue after exercise [19].

Although this study confirmed that 28-day SYNKEFIR™ supplementation can significantly improve exercise endurance performance, this supplement did not seem to have a notable effect on the gut microbiota (Figure 3). This may require a longer supplement period or a higher dose. In our previous study with a mouse model, compared to the five-dose intervention dose in this trial, a longer period could significantly increase the bacterial fraction [19]. Another study explored the impact of daily yogurt supplementation on the composition of the gut microbiota. It was found that the gut microbiota began to change after 7 days of yogurt supplementation, but no obvious effect appeared until the 42nd day [33]. However, supplementation with yogurt containing probiotics for two weeks was able to reduce inflammation after exhaustive exercise and slow the production of oxidative stress, which seems to be a factor in improving endurance performance [34]. In addition, in the current study on blood biochemistry, hematology, and human body composition, it was found that continuous supplementation with SYNKEFIR™ for 28 days does not cause any harm or burden to the human body.

## 4. Materials and Methods

### 4.1. SYNKEFIR™ and Placebo Preparation

SYNKEFIR™ was prepared by inoculating pasteurized 9.2% reconstituted skim milk with lyophilized kefir starter and then fermenting the milk at 37 °C for 16 h. Sixteen percent (16% *w/w*) of maltodextrin, which was purchased from PT. Sorini Agro Asia Corporindo TBK (Jawa Timur, Indonesia), was added to the fermented milk, mixed well, pasteurized at 100 °C for 30 min and spray-dried. Placebo was prepared with the same materials and procedure without starter added but directly acidified with about 0.63% of lactic acid, the same amount of lactic acid as SYNKEFIR after fermentation measured by titration. The spray-drying procedure for placebo was the same as SYNKEFIR™. Both products were indistinguishable from each other on appearance and taste. The samples were packed in aluminum pouch every 20 g and stored at 4 °C until further use. Each 100 g of SYNKEFIR™ or placebo contained 15 g of protein, and 78.5 g of carbohydrates, with a total of 374 kilocalories. The SYNKEFIR™ starter culture used for inoculation was composed of specific lactic acid bacteria strains, including *L. paracasei* DSM 32785 (LPC12), *L. rhamnosus* DSM 32786 (LRH10), *L. helveticus* DSM 32787 (LH43), *L. fermentum* DSM 32784 (LF26), and *S. thermophilus* DSM 32788 (ST30), as previously described [19]. All strains were isolated from traditional kefir and were provided by SYNBIO TECH INC. (Kaohsiung, Taiwan).

### 4.2. Subjects

**Figure 4:**
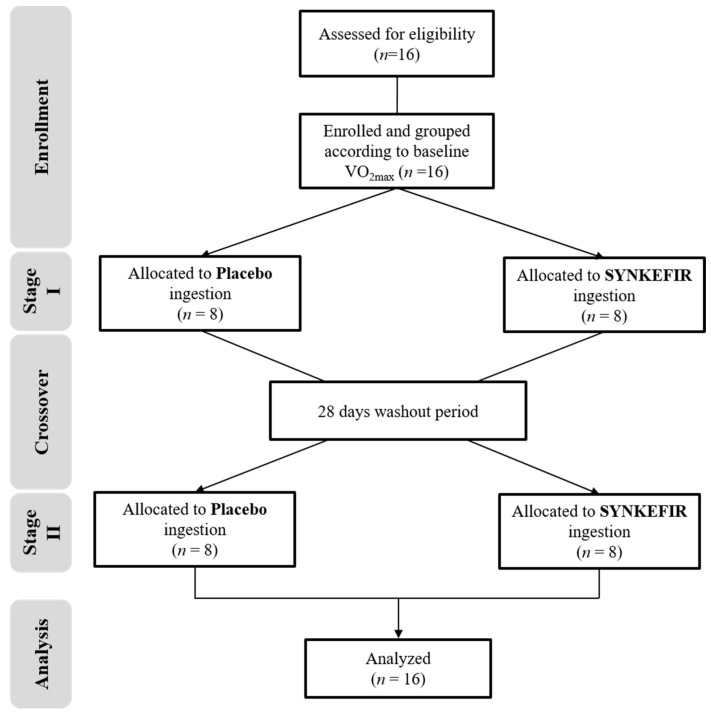
Experimental procedure description.

This study included those with ages between 20 and 30 years old who were healthy and untrained males and excluded the volunteers who had smoking or drinking habits; engaged in the long-term use of nutritional supplements or drugs; or had food allergies, liver or kidney dysfunction, cardiovascular disease, or other chronic diseases. All volunteers were prohibited from taking probiotics, prebiotic fermented products (yogurt or cheese), vitamins, minerals, herbal extracts, dietary supplements for exercise, or antibiotics to avoid unnecessary interference during the experiment. The study was reviewed and approved by the Taipei Medical University Joint Institutional Review Board (Taipei, Taiwan; No. 201611024). After explaining the experimental process and content in detail, all volunteers provided written informed consent before participating. The experimental procedure description is shown in Figure 4, and the basic demographic profiles and characteristics of the subjects are shown in Table 6.

### 4.3. Experimental Design

This test used a double-blind crossover design and was supplemented for 28 days. The 16 subjects were divided into Placebo or SYNKEFIR™ group in a balanced order according to each individual’s initial maximal oxygen uptake. Enrolled subjects were requested to intake 1 pouch of placebo or SYNKEFIR™, which was recommended to rehydrated with 200 mL of water before ingestion, every morning. After the intervention, there were 28 days of wash-out, during which time the subjects did not receive further intervention. This was followed by a second 28 days replacement therapy intervention cycle (subjects who received the placebo during the first cycle received SYNKEFIR™, and vice versa). All subjects only supplemented during the experimental, and did not involve any exercise training.

**Figure 5:**
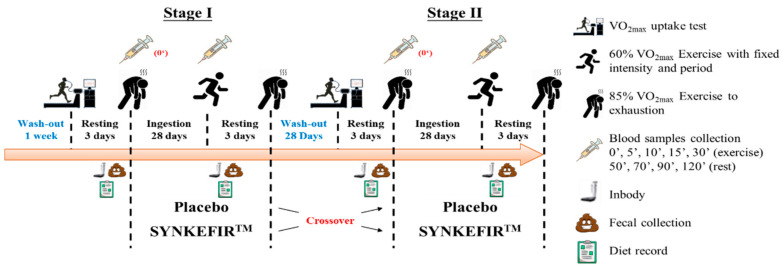
Experimental design.

Before each stage of interventions, we measured the body composition, common blood biochemical parameters, and exercise endurance capacity of the subjects and collected fecal samples. Then, after four consecutive weeks of supplementation, all subjects were asked to test their exercise fatigue biochemical value and exercise endurance performance and body composition, and to collected fecal samples. In addition, to confirm that the exercise performance of each group of subjects would not be affected by diet, during the supplement and test periods, all subjects recorded their diets, and professional nutritionists analyzed and calculated their daily carbohydrate, protein, fat, and total caloric intake levels, there has no significantly different on their daily nutritional intake and calories. The detailed experimental procedure is illustrated in Figure 5**.**

### 4.4. VO_2max_ and Endurance Performance Test

To evaluate the maximum oxygen consumption and exercise performance, we used a treadmill (Pulsar, h/p/cosmos, Nussdorf-Traunstein, Germany) and an automatic breathing analyzer (Vmax 29c, Sensor Medics, Yorba Linda, CA, USA). In addition, a polar heart rate device was used to monitor the heart rate (HR). The maximum oxygen uptake assessment method that we used was previous described in [26]. The speed range of the treadmill was set to 7.2 km/h and increased by 1.8 km/h every 2 min until fatigue, according to Bruce’s protocol [35]. When the breathing exchange rate (the volume ratio of carbon dioxide produced to oxygen consumed, VCO_2_/VO_2_) was higher than 1.10 and reached the maximum heart rate (maximum heart rate = 220 − age), oxygen consumption was considered to be maximum. The three highest VO_2max_ peak were averaged to obtain the VO_2max_ values of the individual volunteers.

The individual basal VO_2max_ values during the pretest were used as a reference to adjust the individually appropriate exercise intensity. Before entering Stage II, all subjects were asked to measure VO_2max_ again to ensure the wash-out period was enough to exclude the interference of treatment difference from Stage I. The subjects performed the maximum endurance test on a treadmill, warmed up to 60% VO_2max_ intensity for 5 min, and then started the endurance running test at 85% VO_2max_ intensity. For the maximum endurance exercise, we used HR and Borg’s rating of perceived exertion every 5 min to monitor the subjects’ physical conditions and continue to record the exercise time to exhaustion. The detailed formula for intensity adjustment was based on that in a previous study [26].

### 4.5. Body Composition

The multi-frequency principle was applied to measure body composition by using a bioelectrical impedance analyzer (BIA) on an InBody 570 device (In-body, Seoul, South Korea). This device can provide frequency shielding of 1, 5, 50, 260, 500, and 1000 kHz within 60 s. To perform the measurements, after the subjects’ palms and soles were removed from the sensors, the subjects stood on the footing electrodes and held the sensing handles with two hands. During the measurements, the subjects kept their arms open and left their bodies at an angle of 30° without speaking or moving. The subjects also fasted for at least 8 h before the test, and the test was performed before measuring the VO_2max_ at each stage.

### 4.6. Clinical Biochemistry and Hematology Analysis

To confirm the basic biochemical parameters and health status of the subjects before each stage of supplementation, the subjects were required to have their blood collected before supplementation and fast for 8 h the previous night. We also analyzed the subjects’ liver function: aspartate transaminase (AST), alanine aminotransferase (ALT), blood urea nitrogen (BUN), albumin (ALB); kidney function: creatinine (CREA), uric acid (UA), total protein (TP); lipid metabolism: free fatty acid (FFA), total cholesterol (TC), triglyceride (TG), high-density lipoprotein (HDL), and low-density lipoprotein (LDL) indicators. In addition, to assess fatigue-related indicators, subjects had to fast for at least 8 h before each 60% VO_2max_ exercise and perform a fixed-intensity challenged with exercise and a recovery period at the indicated time points, including the baseline (0), 5 (E5), 10 (E10), 15 (E15), and 30 (E30) minutes, respectively, with 20 (R20), 40 (R40), 60 (R60), and 90 (R90) minutes used in the exercise phase. To evaluate the physiological fitness of lactate, ammonia (NH_3_), glucose, and creatinine kinase (CK), all biochemical indices were assessed using an autoanalyzer (Hitachi 7060, Tokyo, Japan). The complete blood count (CBC) profiles (MindrayBC-2800Vet, Shenzhen, China) were also analyzed before each stage of supplementation.

### 4.7. Detection of Lactobacillus spp., Bifidobacterium spp., Clostridium spp., and Bacteroides spp. by Quantitative PCR

The fresh fecal sample was resuspended with sterile 0.85% saline at a ratio of 1:9 and vortexed until a homogenous suspension was obtained. A 200 μL homogenized suspension was then washed twice with 1 mL phosphate buffered saline (PBS) and centrifuged at 12,000 rpm for 5 min, and 1 mL of the supernatant was discarded. Glass beads (product No. 11079110, 1 mm; BioSpec Products, Bartlesville, OK, USA) were added to the 200 μL washed fecal suspension and vortexed at 3000 rpm for 30 min to disrupt the bacteria cells. Fecal DNA was subsequently extracted using a Genomic DNA mini kit (Geneaid, GB300, New Taipei, Taiwan) following the manufacturer’s instructions. The *Lactobacillus* spp., *Bifidobacterium* spp., *Clostridium* spp., and *Bacteroides* spp. in the fecal samples were then enumerated by quantitative real-time PCR (qPCR). The qPCR analyses were performed using QuantStudioTM 3 real-time PCR systems (Thermo Fisher SCIENTIFIC, Grand Island, NY, USA). Each reaction mixture (20 μL) was composed of 10 μL PowerUp™ SYBR™ Green Master Mix (Applied Biosystems, Austin, TX, USA), 2 μL of 10 μM specific primers (forward and reverse) (Table 7), and 1 μL of appropriate diluted template DNA. For determination of the bacterial count of *Lactobacillus* spp., *Bifidobacterium* spp., *Clostridium* spp., and *Bacteroides* spp. present in each sample, the fluorescent signals detected from two or three serial dilutions in the linear range of the assay were averaged and compared to a standard curve generated with DNA extracted from the corresponding standard strains, including *Lactobacillus casei* BCRC10697, *Bifidobacterium animals* subsp. *lactis* BCRC17394, *Clostridium sphenoides* BCRC14515, and *Bacteroides fragilis* BCRC10620 in the same experiment [36].

### 4.8. Statistical Analysis

All data are expressed as the mean ± SD. Statistical analyses were performed in GraphPad Prism (version 7.04, GraphPad Software Inc., San Diego, CA, USA). Intergroup differences were analyzed by Student’s unpaired t-test. Mann-Whitney U test was used for comparison non-parametric data, including fat mass, ratio of the body composition change, ratio of neutrophil to lymphocyte (NLR), and ratio of platelet to lymphocyte (PLR). Specifically, statistical significance of Time to Exhaustion (Figure 1) and gut microbiota (Figure 3) were analyzed using two-way repeated measures ANOVA post hoc Bonferroni test. Differences were considered statistically significant at *p* < 0.05. The required sample sizes for clinical trials based on this change were calculated using the Harvard calculator (http://hedwig.mgh.harvard.edu/sample_size/size.html, accessed on 14 December 2020), assuming parallel design with 0.05 significance level, the change SD, power of 0.8, standard deviation of the difference with 3.2. At least a total of 15 patients will enter this two-treatment crossover study.

## 5. Conclusions

In the present study, although supplementation with SYNKEFIR™ for 28 consecutive days did not significantly change the gut microbiota, we found that SYNKEFIR™ effectively improved exercise endurance performance, reduced the production of lactic acid after exercise, and accelerated recovery. In addition, this study also confirmed that SYNKEFIR™ does not cause any harm or burden to the human body.

## Figures and Tables

**Table 1:** Biochemistry analysis of the subjects before ingestion.

Baseline	Placebo (*n* = 16)	SYNKEFIR™ (*n* = 16)
Lactate (mmol/L)	2.23 ± 0.49	2.14 ± 0.67
NH_3_ (μmol/L)	72 ± 32	94 ± 35
CPK (U/L)	148 ± 45	200 ± 124
Glucose (mg/dL)	91 ± 11	89 ± 9
FFA (mmol/L)	0.50 ± 0.26	0.45 ± 0.21
AST (U/L)	19 ± 4	18 ± 8
ALT (U/L)	23 ± 12	22 ± 10
TC (mg/dL)	211 ± 32	191 ± 22 *
TG (mg/dL)	81 ± 24	83 ± 26
HDL (mg/dL)	60 ± 6	57 ± 6
LDL (mg/dL)	112 ± 21	106 ± 14
BUN (mg/dL)	14.3 ± 2.9	12.9 ± 1.9
CREA (mg/dL)	1.16 ± 0.09	1.13 ± 0.11
UA (mg/dL)	5.64 ± 0.99	5.63 ± 1.17
TP (g/dL)	7.48 ± 0.43	7.36 ± 0.33
ALB (g/dL)	5.10 ± 0.33	5.11 ± 0.24

Data are shown as the mean ± SD, and intergroup differences were analyzed using Student’s unpaired *t*-test. * *p* < 0.05. NH_3_, blood ammonia; CK, creatine kinase; FFA, free fatty acid; AST, aspartate aminotransferase; ALT, alanine aminotransferase; BUN, blood urea nitrogen; CREA, creatine; UA, uric acid; TP, total protein; TC, total cholesterol; TG, triacylglycerol. HDL, high-density lipoprotein; LDL, low-density lipoprotein.

**Table 2:** Blood count profiles of the subjects.

Baseline (CBC)	Placebo (*n* = 16)	SYNKEFIR™ (*n* = 16)
WBC (cells/mcL)	7309 ± 1800	6824 ± 1543
Neutrophils (%)	64.0 ± 12.4	59.1 ± 6.8
Lymphocytes (%)	28.7 ± 10.8	31.5 ± 6.4
Monocytes (%)	5.0 ± 1.5	5.9 ± 1.3
Eosinophil (%)	1.7 ± 1.5	2.8 ± 1.9
Basophil (%)	0.5 ± 0.2	0.6 ± 0.3
Platelet (10^3^/mcL)	297 ± 53	289 ± 59
NLR	3.21 ± 3.24	2.00 ± 0.66
PLR	174.8 ± 99.7	140.5 ± 31.8

Data are shown as the mean ± SD. Intergroup differences were analyzed using Student’s unpaired *t*-test. For NLR and PLR, the statistical significance was analyzed using Mann-Whitney U test. WBC, white blood cell; NLR, the ratio of neutrophil to lymphocyte ratio; PLR, the ration of platelet to lymphocyte.

**Table 3:** Biochemistry analysis of the subjects after ingestion.

		Placebo (*n* = 16)		SYNKEFIR™ (*n* = 16)		
Time Point	0′	E30′	R90′	0′	E30′	R90′
FFA (mmol/L)	0.63 ± 0.26	0.73 ± 0.30	0.90 ± 0.22	0.55 ± 0.18	0.58 ± 0.23	0.82 ± 0.20
AST (U/L)	26 ± 8	25 ± 7	22 ± 5	27 ± 11	27 ± 12	26 ± 11
ALT (U/L)	28 ± 14	28 ± 15	24 ± 9	32 ± 22	34 ± 20	31 ± 21
TC (mg/dL)	209 ± 33	224 ± 39	207 ± 30	204 ± 33	207 ± 23	195 ± 23
TG (mg/dL)	87 ± 36	103 ± 36	75 ± 29	84 ± 19	95 ± 21	76 ± 22
HDL (mg/dL)	60 ± 9	63 ± 11	59 ± 5	63 ± 16	64 ± 10	62 ± 8
LDL (mg/dL)	111 ± 15	117 ± 26	110 ± 16	110 ± 18	112 ± 20	105 ± 14
BUN (mg/dL)	13.9 ± 2.4	14.2 ± 2.8	14.6 ± 3.1	13.7 ± 3.0	13.9 ± 2.9	14.2 ± 3.3
CREA (mg/dL)	1.19 ± 0.13	1.29 ± 0.13	1.24 ± 0.13	1.16 ± 0.12	1.23 ± 0.12	1.20 ± 0.10
UA (mg/dL)	5.74 ± 1.35	6.06 ± 1.33	6.10 ± 1.36	5.66 ± 1.17	6.01 ± 1.15	5.95 ± 1.14
TP (g/dL)	7.58 ± 0.65	7.93 ± 0.87	7.41 ± 0.48	7.83 ± 0.75	8.16 ± 1.00	7.55 ± 0.58
ALB (g/dL)	5.1 ± 0.4	5.3 ± 0.5	5.0 ± 0.4	5.3 ± 0.5	5.4 ± 0.6	5.1 ± 0.5

Data are shown as the mean ± SD. Statistical differences between groups at the same collection time points were analyzed by Student’s unpaired *t*-test. E: exercise; R: rest.

**Table 4:** Blood count profiles of the subjects after ingestion.

CBC	Placebo (*n* = 16)			SYNKEFIR™ (*n* = 16)		
Time Point	0′	E30′	R90′	0′	E30′	R90′
WBC (cells/mcL)	6817 ± 1556	9144 ± 1693	8188 ± 2012	7307 ± 2066	8797 ± 2295	7812 ± 2136
Neutrophils (%)	59.36 ± 8.17	55.60 ± 10.66	64.29 ± 7.86	59.92 ± 7.32	57.68 ± 8.07	64.49 ± 8.11
Lymphocytes (%)	31.27 ± 7.23	35.88 ± 10.16	27.49 ± 7.24	30.61 ± 6.95	33.51 ± 7.31	27.33 ± 7.43
Monocytes (%)	5.44 ± 1.70	5.67 ± 1.88	5.51 ± 1.33	5.94 ± 1.92	5.72 ± 1.65	5.69 ± 1.44
Eosinophil (%)	3.36 ± 0.84	2.26 ± 1.34	2.17 ± 1.22	2.92 ± 1.09	2.54 ± 1.20	2.01 ± 0.91
Basophil (%)	0.6 ± 0.2	0.6 ± 0.2	0.5 ± 0.2	0.6 ± 0.3	0.6 ± 0.2	0.5 ± 0.1
Platelet (10^3^/mcL)	281 ± 66	320 ± 69	284 ± 62	286 ± 52	308 ± 50	285 ± 51
NLR	2.06 ± 0.78	1.76 ± 0.86	2.60 ± 1.08	2.14 ± 0.88	1.88 ± 1.09	2.67 ± 1.30
PLR	140.0 ± 38.3	109.6 ± 42.0	144.2 ± 59.0	137.9 ± 34.0	112.9 ± 31.2	147.0 ± 42.7

Data are shown as the mean ± SD. Statistical significance between Placebo and SYNKEFIR™ groups at same collection time points were analyzed by Student’s unpaired *t*-test. For NLR ad PLR, Mann-Whitney U test was applied to analyze the statistical differences between treatment groups. WBC, white blood cell; NLR, neutrophil/lymphocyte ratio; PLR, platelet/lymphocyte ratio.

**Table 5:** Body composition of subjects before and after ingestion.

Body Composition	Placebo (*n* = 16)			SYNKEFIR™ (*n* = 16)		
	Before Ingestion	After Ingestion	Change	Before Ingestion	After Ingestion	Change
Body Weight (g)	70.69 ± 9.52	70.75 ± 9.69	0.06 ± 1.15	72.38 ± 10.84	72.33 ± 10.68	−0.04 ± 1.07
BMI (kg/m^2^)	23.66 ± 2.83	23.67 ± 2.84	0.01 ± 0.41	24.00 ± 2.95	24.56 ± 3.92	0.56 ± 2.43
Fat Mass (%)	20.14 ± 6.86	19.91 ± 7.03	−0.23 ± 1.28	21.01 ± 7.07	20.31 ± 6.26	−0.69 ± 1.40
Muscle Mass (kg)	31.76 ± 3.21	32.02 ± 3.39	0.26 ± 0.79	32.14 ± 3.38	32.48 ± 3.64	0.33 ± 0.75

Data are shown as the mean ± SD. Statistical significance between Placebo and SYNKEFIR™ groups on body weight, BMI, and muscle mass were analyzed using Student’s unpaired *t*-test, and Mann-Whitney U test was used for statistical comparison on fat mass. The changes on body weight, BMI, fat mass, and muscle mass were calculated as the difference between after and before ingestion and statistical significance was analyzed using the Mann-Whitney U test. BMI, body mass index.

**Table 6:** Background data of the subjects.

Group	Placebo in Stage I	SYNKEFIR™ in Stage I
Subjects	*n* = 8	*n* = 8
Age	25.6 ± 4.1	24.6 ± 2.8
Height (cm)	172.5 ± 5.0	174.6 ± 5.2
VO_2max_ (mL/kg/min)	46.6 ± 8.2	47.3 ± 6.5

Data are expressed as the mean ± SD. There were no significant differences in the basic information data between the two groups.

**Table 7:** Primers used in the quantitative real-time PCR.

Target Bacterial Group	Primer Sequence (5′ → 3′)	Predicted Product Size (bp)
*Lactobacillus* genus [37]	F: AGCAGTAGGGAATCTTCCA R: CACCGCTACACATGGAG	341
*Bifidobacterium* genus [37]	F: GGGTGGTAATGCCGGATG	442
	R: TAAGCGATGGACTTTCACACC	
*Clostridium* genus [37]	F: CGGTACCTGACTAAGAAGC	429
	R: AGTTTYATTCTTGCGAACG	
*Bacteroides* genus [38]	F: GGGTTTAAAGGGAGCGTAGG	116
	R: CTACACCACGAATTCCGCCT	

F, forward; R, reverse; bp, base pairs.

## Data Availability

The data presented in this study are available within the article.

## References

[1] Wan J.J., Qin Z., Wang P.Y., Sun Y., Liu X. (2017). Muscle fatigue: General understanding and treatment. Exp. Mol. Med..

[2] Finsterer J. (2012). Biomarkers of peripheral muscle fatigue during exercise. BMC Musculoskelet. Disord..

[3] Chi A., Li H., Kang C., Guo H., Wang Y., Guo F., Tang L. (2015). Anti-fatigue activity of a novel polysaccharide conjugates from Ziyang green tea. Int. J. Biol. Macromol..

[4] Rivera-Brown A.M., Frontera W.R. (2012). Principles of exercise physiology: Responses to acute exercise and long-term adaptations to training. Am. Acad. Phys. Med. Rehabil..

[5] Proia P., Di Liegro C.M., Schiera G., Fricano A., Di Liegro I. (2016). Lactate as a metabolite and a regulator in the central nervous system. Int. J. Mol. Sci..

[6] Chen W.C., Huang W.C., Chiu C.C., Chang Y.K., Huang C.C. (2014). Whey protein improves exercise performance and biochemical profiles in trained mice. Med. Sci. Sports Exerc..

[7] Allen J.M., Mailing L.J., Niemiro G.M., Moore R., Cook M.D., White B.A., Holscher H.D., Woods J.A. (2018). Exercise alters gut microbiota composition and function in lean and obese humans. Med. Sci. Sports Exerc..

[8] Clarke S.F., Murphy E.F., O’Sullivan O., Lucey A.J., Humphreys M., Hogan A., Hayes P., O’Reilly M., Jeffery I.B., Wood-Martin R. (2014). Exercise and associated dietary extremes impact on gut microbial diversity. Gut.

[9] Mohr A.E., Jäger R., Carpenter K.C., Kerksick C.M., Purpura M., Townsend J.R., West N.P., Black K., Gleeson M., Pyne D.B. (2020). The athletic gut microbiota. J. Int. Soc. Sports Nutr..

[10] Jung Y.P., Earnest C.P., Koozehchian M., Cho M., Barringer N., Walker D., Rasmussen C., Greenwood M., Murano P.S., Kreider R.B. (2017). Effects of ingesting a pre-workout dietary supplement with and without synephrine for 8 weeks on training adaptations in resistance-trained males. J. Int. Soc. Sports Nutr..

[11] LeBlanc J.G., Chain F., Martín R., Bermúdez-Humarán L.G., Courau S., Langella P. (2017). Beneficial effects on host energy metabolism of short-chain fatty acids and vitamins produced by commensal and probiotic bacteria. Microb. Cell Fact..

[12] Mach N., Fuster-Botella D. (2017). Endurance exercise and gut microbiota: A review. J. Sport Health Sci..

[13] Bensmira M., Nsabimana C., Jiang B. (2010). Effects of fermentation conditions and homogenization pressure on the rheological properties of Kefir. Food Sci. Technol..

[14] Serafini F., Turroni F., Ruas-Madiedo P., Lugli G.A., Milani C., Duranti S., Zamboni N., Bottacini F., van Sinderen D., Margolles A. (2014). Kefir fermented milk and kefiran promote growth of *Bifidobacterium bifidum* PRL2010 and modulate its gene expression. Int. J. Food Microbiol..

[15] Wszolek M., Tamime A., Muir D., Barclay M. (2001). Properties of kefir made in Scotland and Poland using bovine, caprine and ovine milk with different starter cultures. LWT Food Sci. Technol..

[16] Altay F., Karbancıoglu-Güler F., Daskaya-Dikmen C., Heperkan D. (2013). A review on traditional Turkish fermented non-alcoholic beverages: Microbiota, fermentation process and quality characteristics. Int. J. Food Microbiol..

[17] Slattery C., Cotter P.D., O’Toole P.W. (2019). Analysis of health benefits conferred by *Lactobacillus* species from kefir. Nutrients.

[18] O’Brien K.V., Stewart L.K., Forney L.A., Aryana K.J., Prinyawiwatkul W., Boeneke C.A. (2015). The effects of postexercise consumption of a kefir beverage on performance and recovery during intensive endurance training. J. Dairy Sci..

[19] Hsu Y.J., Huang W.C., Lin J.S., Chen Y.M., Ho S.T., Huang C.C., Tung Y.T. (2018). Kefir supplementation modifies gut microbiota composition, reduces physical fatigue, and improves exercise performance in mice. Nutrients.

[20] Barton W., Penney N.C., Cronin O., Garcia-Perez I., Molloy M.G., Holmes E., Shanahan F., Cotter P.D., O’Sullivan O. (2018). The microbiome of professional athletes differs from that of more sedentary subjects in composition and particularly at the functional metabolic level. Gut.

[21] Samuel B.S., Shaito A., Motoike T., Rey F.E., Backhed F., Manchester J.K., Hammer R.E., Williams S.C., Crowley J., Yanagisawa M. (2008). Effects of the gut microbiota on host adiposity are modulated by the short-chain fatty-acid binding G protein-coupled receptor, Gpr41. Proc. Natl. Acad. Sci. USA.

[22] Clark A., Mach N. (2017). The crosstalk between the gut microbiota and mitochondria during exercise. Front. Physiol..

[23] Leite G.S.F., Resende Master Student A.S., West N.P., Lancha A.H. (2019). Probiotics and sports: A new magic bullet?. Nutrition.

[24] Lin C.L., Hsu Y.J., Ho H.H., Chang Y.C., Kuo Y.W., Yeh Y.T., Tsai S.Y., Chen C.W., Chen J.F., Huang C.C. (2020). *Bifidobacterium longum* subsp *longum* OLP-01 supplementation during endurance running training improves exercise performance in middle- and long-distance runners: A double-blind controlled trial. Nutrients.

[25] Chen Y.M., Wei L., Chiu Y.S., Hsu Y.J., Tsai T.Y., Wang M.F., Huang C.C. (2016). *Lactobacillus plantarum* TWK10 Supplementation improves exercise performance and increases muscle mass in mice. Nutrients.

[26] Huang W.C., Lee M.C., Lee C.C., Ng K.S., Hsu Y.J., Tsai T.Y., Young S.L., Lin J.S., Huang C.C. (2019). Effect of *Lactobacillus plantarum* TWK10 on exercise physiological adaptation, performance, and body composition in healthy humans. Nutrients.

[27] Salarkia N., Ghadamli L., Zaeri F., Sabaghian Rad L. (2013). Effects of probiotic yogurt on performance, respiratory and digestive systems of young adult female endurance swimmers: A randomized controlled trial. Med. J. Islam. Repub. Iran..

[28] Osnes J.B., Hermansen L. (1972). Acid-base balance after maximal exercise of short duration. J. Appl. Physiol..

[29] Hall M.M., Rajasekaran S., Thomsen T.W., Peterson A.R. (2016). Lactate: Friend or foe. Am. Acad. Phys. Med. Rehabil..

[30] Hermansen L., Maehlum S., Pruett E.D.R., Vaage O., Waldum H., Wessel-Aas T. (1975). Lactate removal at rest and during exercise. Metabolic Adaptation to Prolonged Physical Exercise.

[31] Chatham J.C. (2002). Lactate—The forgotten fuel!. J. Physiol..

[32] Coqueiro A.Y., de Oliveira Garcia A.B., Rogero M.M., Tirapegui J. (2017). Probiotic supplementation in sports and physical exercise: Does it present any ergogenic effect?. Nutr. Health.

[33] Lisko D.J., Johnston G.P., Johnston C.G. (2017). Effects of dietary yogurt on the healthy human gastrointestinal (GI) microbiome. Microorganisms.

[34] Mazani M., Nemati A., Amani M., Haedari K., Mogadam R.A., Baghi A.N. (2018). The effect of probiotic yoghurt consumption on oxidative stress and inflammatory factors in young females after exhaustive exercise. J. Pak. Med. Assoc..

[35] Bruce R.A., Kusumi F., Hosmer D. (1973). Maximal oxygen intake and nomographic assessment of functional aerobic impairment in cardiovascular disease. Am. Heart J..

[36] Matsuki T., Watanabe K., Fujimoto J., Kado Y., Takada T., Matsumoto K., Tanaka R. (2004). Quantitative PCR with 16S rRNA-gene-targeted species-specific primers for analysis of human intestinal bifidobacteria. Appl. Environ. Microbiol..

[37] Kook S.Y., Kim Y., Kang B., Choe Y.H., Kim Y.H., Kim S. (2018). Characterization of the fecal microbiota differs between age groups in Koreans. Intest. Res..

[38] Layton A., McKay L., Williams D., Garrett V., Gentry R., Sayler G. (2006). Development of bacteroides 16S rRNA gene TaqMan-based real-time PCR assays for estimation of total, human, and bovine fecal pollution in water. Appl. Environ. Microbiol..

